# Valorisation of Rabbit Biodiversity for Meat Production: Live Performance, Carcass Traits, Meat Quality and Muscle Fibre Characteristics of Different Rabbit Genotypes

**DOI:** 10.3390/ani16060937

**Published:** 2026-03-17

**Authors:** Antonella Dalle Zotte, Cecilia Mugnai, Bianca Palumbo, Marco Cullere

**Affiliations:** 1 Department of Animal Medicine, Production and Health, University of Padova, Agripolis, Viale dell’Università 16, 35020 Legnaro, Padova, Italy; bianca.palumbo@unipd.it (B.P.); marco.cullere@unipd.it (M.C.); 2 Department of Veterinary Sciences, University of Turin, Largo Paolo Braccini, 2, 10095 Grugliasco, Torino, Italy; cecilia.mugnai@unito.it

**Keywords:** rabbit, breed, genotype, biodiversity, sustainable development, live performance, carcass traits, muscle fibres, meat quality, nutrition

## Abstract

Valorisation of rabbit biodiversity plays a significant role in enhancing production by preserving genetic diversity, which is crucial for maintaining adaptability and resilience in rabbit populations, thereby supporting sustainable development and conservation efforts. With this in mind, the present research aimed at comparing live performance, carcass traits, meat quality and muscle fibre characteristics of different rabbit genotypes. Forty-five weaned rabbits (15 commercial hybrids—C; 15 Burgundy Fawn crosses—BF; 15 Vienna Blue crosses—VB) were farmed until slaughter (n = 15 replicated cages/rabbit genotype). The slaughter age was scheduled when all genotypes reached the same live weight (approx. 2800 g). After slaughtering and carcass dissection, the hind legs and *longissimus lumborum* muscles were excised and subjected to different evaluations. Hind legs were exploited for physicochemical analyses, while *longissimus lumborum* muscles were used for physical evaluations and for fibre typing, morphometric traits and enzymatic activity. As a direct response to the experimental design, results highlighted that the three genotypes exhibited different slaughter ages. Commercial hybrids displayed the fastest growth cycle, but they showed an efficiency comparable to that of VB crosses (*p* > 0.05). Genotypes displayed some differences in carcass characteristics, namely perirenal fat (*p* < 0.01) and hind leg weight (*p* < 0.05). The physical characteristics of meat were overall similar in the three genotypes, except for *biceps femoris* L*, which showed the highest value in the BF group. Meat chemical composition differed depending on the genotype, with BF rabbits having the highest ether extract (*p* < 0.05) content. The three genotypes displayed an overall similar fatty acid profile with some minor differences: VB rabbits had the highest C18:2 *n*-6 proportion (*p* < 0.01) and thus *n*-6/*n*-3 (*p* < 0.05). Cholesterol content was the highest in C rabbits (*p* < 0.05). Overall, the present research highlighted that BF and VB genotypes provided interesting potentialities which would be further valorised in rural farming conditions, given their higher resilience and adaptability than commercial hybrids.

## Simple Summary

Rabbit meat production increasingly depends on a few high-performance rabbit genotypes, which can reduce genetic diversity and limit the adaptability of production systems, especially in rural regions where most of the demographic growth is occurring and where food security is a challenge. To address this concern and support sustainable farming practices, the present research studied the growth, carcass traits, meat quality, and muscle characteristics of three different rabbit genotypes raised under the same conditions and slaughtered at the same weight. Commercial rabbits grew fastest, but the alternative genotypes, Burgundy Fawn and Vienna Blue, showed comparable carcass yields and largely similar meat quality traits. Some differences were found in fat content, bone strength, and fatty acid composition, which can influence both nutrition and consumer preferences. These results show that local or less selected rabbit types can produce good-quality meat and may represent promising alternatives in diversified production systems. By highlighting the potential of different rabbit types, this study supports the conservation and practical use of genetic diversity in rabbit farming, especially in rural areas.

## 1. Introduction

In recent decades, livestock production systems have increasingly focused on a limited number of highly specialised genotypes, primarily selected for productivity and efficiency. While this approach has contributed to improvements in production performance, it has also raised concerns regarding the global erosion of animal genetic diversity (e.g., risk of epizootics and production instability), reduced adaptability to environmental challenges, and increased vulnerability to changing climatic and socio-economic conditions [1,2]. In this context, the valorisation of local and alternative genetic resources represents a key strategy for promoting sustainable animal production systems that balance productivity, resilience, and biodiversity conservation [3]. Rabbit production is a relevant example of this dual challenge.

Commercial rabbit meat production is largely based on hybrid lines selected for rapid growth (which determines a prevalence of glycolytic metabolism), yields and feed efficiency under intensive conditions [4]. However, such genotypes may be less suited to extensive or semi-intensive farming systems, where robustness, adaptability, and product differentiation are increasingly valued [5]. Conversely, local rabbit breeds and traditional genotypes, although generally characterised by slower growth rates, often display higher resilience to environmental stressors and diseases, making them potentially advantageous in low-input and rural production systems [6]. The preservation and utilisation of rabbit biodiversity have therefore gained growing attention in recent years.

Several studies have investigated the productive performance, carcass characteristics, and meat quality of local rabbit breeds or alternative genotypes, highlighting both their strengths and limitations when compared to commercial hybrids [7,8,9].

Meat quality traits, including physicochemical properties, lipid composition, and cholesterol content, are of particular interest, as they influence both consumer acceptance and the nutritional value of rabbit meat [10]. Moreover, muscle fibre composition and enzymatic activity have emerged as important determinants of meat quality and growth potential, providing deeper insight into the biological mechanisms underlying genotype-related differences [11].

Despite this growing body of research, results remain partially inconsistent. Some authors report inferior carcass yield and fat deposition in local or non-selected genotypes compared with commercial hybrids [12], while others highlight comparable or even superior meat quality traits, particularly in terms of fatty acid profile and protein content [13]. These contrasting findings represent a significant research gap and underline the need for further comparative studies conducted under standardised conditions, especially those integrating productive performance, carcass traits, meat quality, and muscle fibre characteristics within the same experimental framework.

The present study, therefore, aimed to compare live performance, carcass traits, meat quality, and muscle fibre characteristics of three rabbit genotypes: commercial hybrid, Burgundy Fawn crosses, and Vienna Blue crosses. By slaughtering animals at a common market weight, the study sought to disentangle genotype-related differences from those related to body size or maturity, hypothesising that this allows the identification of key specificities in studied local breeds and commercial hybrids. The results will contribute to a better understanding of the productive and qualitative potential of alternative rabbit genotypes and support the valorisation of rabbit biodiversity as a viable strategy for sustainable meat production, particularly in rural and low-input farming systems.

## 2. Materials and Methods

### 2.1. Experimental Design, Rabbit Farming, Slaughtering, Carcass Dissection, Samples Preparation and Physical Measurements

For this study, n = 45 weaned rabbits were used, allocated as follows: 15 conventional hybrids (n = 12 M and n = 3 F) from conventional breeding (C), and 30 from organic breeding, of which 15 Burgundy Fawn (BF: 7 M and 8 F) from paternal origin and 15 Vienna Blue (VB: 7 M and 8 F) from paternal origin. The subjects from the organic farm were weaned at an age ranging from 36 to 47 days, while the group (C) was weaned at 38 days.

All rabbits were then placed in individual cages with slatted floors and nipple drinkers. Cages were located in a fattening building, randomised in order to avoid the presence of the same breed in two consecutive cages, with a natural ventilation system and natural lighting, in accordance with the directives of the EC Council 1999 [14] and the AIA [15]. The feeders were filled with a standard pellet diet which was the same for all animals (900 g/kg dry matter, 144 g/kg crude protein, 45 g/kg ether extract, 84 g/kg ash, 16.8 g/kg Ca, 6.4 g/kg P, 16.4 MJ/kg gross energy; values are expressed on the as is basis). Feeders were checked daily, and the residues were weighed once a week (included in feed efficiency calculations). The animals were weighed twice a week until they reached 2800 ± 130 g (fixed slaughter weight). During the experiment, there was no rabbit mortality.

Once rabbits reached their slaughter age, they were first weighed (slaughter weight), then stunned and slaughtered at a commercial abattoir. Immediately after slaughter, a small section of the left *longissimus lumborum* (LL) muscle was removed, weighed, and frozen in isopentane cooled by liquid nitrogen and stored at −80 °C for subsequent evaluation of morphometric traits and fibre type distribution [16], as well as enzymatic activity [17].

The carcasses were then left in a cold room at 4 °C for 24 h. At 24 h postmortem, the pHu at the level of the right LL and *biceps femoris* and the colour (CIE L*a*b*) on the same muscles were measured twice on each individual carcass, by using an Ingold electrode (406 M3; Mettler Toledo, Milan, Italy) and a colourimeter (Chromameter CR100, Minolta, Tokyo, Japan), respectively. Being pH and colour measurements conducted at a constant temperature and at consistent post-mortem time points, this ensured repeatability. The head, thymus, trachea, heart, and lungs, liver, and kidneys were then removed from the refrigerated carcass and weighed, and the remaining reference carcass was weighed as described by Blasco et al. [18]. The reference carcass was then defatted, i.e., the interscapular, perirenal, and deposit fat present in the abdominal and inguinal areas and around the neck (other fat) was removed. The loin section from the first lumbar vertebra to the last lumbar vertebra inclusive, and the left and right hind legs were then separated [18]. The right hind leg was deboned to calculate the meat/bone ratio: (leg weight − bone weight)/bone weight.

### 2.2. Physicochemical Evaluations

Immediately after deboning, the meat of the right hind leg was ground at 10,000 rpm for 10 s (Grindomix GM200, Retsch, Haan, Germany), freeze-dried, and used to determine the dry matter (method 934.01), ash (942.05), and ether extract (method 991.36) contents. Protein content, including glucidic molecules and their catabolites (0.25%), was calculated by difference [19]. Values were expressed as g/100 g meat. The Heme iron content was analysed by applying the method described by Hornsey [20], and values were expressed as mg/kg meat.

Freeze-dried samples were also used for lipid extraction [21] and fatty acid determination by gas chromatography using the automated apparatus CE 8000 Top (ThermoQuest Italia S.p.A., Milan, Italy) equipped with a flame ionisation detector and an Omegawax^®^ 250 type capillary column (30 m × 0.25 mm ID) (Sigma-Aldrich, St. Louis, MI, USA). Fatty acids were expressed as % of total Fatty Acids Methyl Esters (FAME). A detailed description of the procedure can be found in Dalle Zotte et al. [22]. In the same publication, a detailed description of the cholesterol determination is also provided. The amount of cholesterol was expressed as mg/kg of meat.

The left femurs (removed from cooked hind legs) were subjected to ta a three-point flexure test by using a universal testing machine (Instron 1000, load rate of 5 mm/min; Instron, Pianezza, Turin). The distance between the two fulcra points supporting the bones was 45 mm. The bones were constantly oriented for testing with their natural convex shape downwards.

### 2.3. Statistical Analysis

During the preliminary check of the datasets, a total of n = 4 rabbits (n = 3 C and n = 1 VB) were excluded from the experiment as they were classified as outliers (rabbits with compromised health status, but not due to factors associated with the experimental design, i.e., genotype and sex effects). Data were then analysed by applying an analysis of variance (ANOVA) using the Proc GLM of version 6 of the SAS software [23] by including the sire genetic origin (SGO: C, VB, BF), sex (S), and their interaction. For live performance, the model included weaning age as a covariate. Least Squares Means were calculated for all the effects included in the model, and the statistical significance of the differences was assessed using orthogonal contrasts. The fixed significance threshold was *p* < 0.05.

## 3. Results

### 3.1. Live Performance

Genotype significantly affected most live performance traits (Table 1): Commercial hybrids (C) showed the highest weaning weight (*p* < 0.001) and reached slaughter weight at a significantly younger age compared with Vienna Blue (VB) and Burgundy Fawn (BF) crosses (*p* < 0.001), confirming their faster growth pattern. Consequently, C rabbits exhibited a shorter fattening period (*p* < 0.001) and a greater average daily gain compared to BF rabbits (*p* < 0.01).

Total feed intake and feed conversion ratio were also influenced by genotype (*p* < 0.01), with BF rabbits displaying the highest feed intake (as the VB group) and the poorest feed efficiency among all groups. Sex had no effect on growth traits, while genotype × sex interactions were limited to average daily feed intake: C: females 139.2 vs. males 141.2 (ns); VB: females 157.8 vs. males 146.8 (*p* < 0.05); BF: females 147.2 vs. males 158.6 (*p* < 0.05).

### 3.2. Carcass Traits

Carcass characteristics were moderately affected by genotype (Table 2). Reference carcass weight and yield did not differ among genotypes. However, perirenal fat weight and incidence on the Reference Carcass (RC) were higher in BF rabbits compared with C and VB rabbits (*p* < 0.05). Hind leg was heavier in VB rabbits compared with BF rabbits (*p* < 0.05), whereas no difference was highlighted for hind leg yield on the RC. Loin incidence on the RC, instead, had a higher incidence on the RC in VB and BF rabbits compared to C ones (*p* < 0.05). Sex did not significantly affect carcass traits, and no relevant genotype × sex interactions were detected.

### 3.3. Physical Traits and Bone Strength

Meat pH values measured 24 h post-mortem were not influenced by genotype or sex in either *biceps femoris* or *longissimus lumborum* muscles (Table 3). However, genotype affected *biceps femoris* lightness (L*), with BF and VB rabbits showing higher values than C rabbits (*p* < 0.05). Femur bone strength differed among genotypes (*p* < 0.05), with VB rabbits exhibiting the highest resistance to fracture stress. Sex influenced bone strength (*p* < 0.05), too, with males showing higher values than females. Instead, no significant genotype × sex interactions were observed.

### 3.4. Muscle Fibre Characteristics

Overall, genotype, sex, and their interactions generally had no significant effect on fibre cross-sectional area, fibre type distribution, or enzymatic activity in the *longissimus lumborum* muscle (Table 4). In fact, the sole significant effect was that of sex on αR fibre cross-sectional area (*p* < 0.05), with males showing larger fibres than females. Enzymatic activity indicators (CS, LDH, LDH/CS) were comparable among genotypes and sexes.

### 3.5. Chemical Composition and Lipid Profile

Genotype affected water and cholesterol contents of the hind leg meat (Table 5). Specifically, BF rabbits showed a higher lipid content than VB, with C being intermediate (*p* < 0.05). Meat of C rabbits exhibited the highest amount of cholesterol (*p* < 0.05), while no difference was observed between VB and BF. Sex influenced water, lipid and cholesterol contents: the meat of female rabbits was characterised by a higher lipid (*p* < 0.001) and cholesterol (*p* < 0.05) than that of males, but by a lower water amount than the meat of male rabbits (*p* < 0.05). Regarding the interaction SGO × S, a significant effect was observed for lipid content of meat: C: females 4.14 vs. males 2.98 (*p* < 0.05); VB: females 3.16% vs. males 3.15% (ns); BF: females 4.78 vs. males 3.04 (*p* < 0.001).

The fatty acid composition of rabbit hind leg meat was influenced by genotype and sex for selected individual fatty acids and lipid ratios (Table 6).

Genotype did not significantly affect total saturated fatty acids (SFA). Among individual SFA, caprylic acid (C8:0) and capric acid (C10:0) were affected by genotype (*p* < 0.01 and *p* < 0.05, respectively): VB had a higher proportion of C8:0 than C and BF, and a higher proportion of C10:0 than BF, but not different from C. No genotype effects were observed for the main SFA, including palmitic (C16:0) and stearic (C18:0) acids. Total monounsaturated fatty acids (MUFA) and single MUFA were not affected by genotype. Genotype significantly influenced linoleic acid (C18:2 *n*-6; *p* < 0.05), with C and VB rabbits showing higher values than BF rabbits. Consequently, the *n*-6/*n*-3 ratio differed among genotypes (*p* < 0.05), with VB rabbits exhibiting the highest values. Total polyunsaturated fatty acids (PUFA) were not affected by genotype.

Sex had the strongest influence on the fatty acids profile of rabbit meat: females showed higher total MUFA percentage (*p* < 0.05) and higher proportions of C14:1, C16:1 and C18:1 *n*-9 (*p* < 0.05), and lower proportion of C18:1 *n*-7 (*p* < 0.01). Males exhibited higher total PUFA (*p* < 0.05) and higher long-chain *n*-3 PUFA proportions, including eicosapentaenoic acid (C20:5 *n*-3; *p* < 0.05) and docosahexaenoic acid (C22:6 *n*-3; *p* < 0.01). As a result, males showed lower MUFA/PUFA ratios than females (*p* < 0.01).

Genotype × sex interactions were not significant for most fatty acid traits, except for the *n*-6/*n*-3 ratio (*p* < 0.05), where differences between sexes were observed within genotypes: C: females 13.0 vs. males 13.6 (ns); VB: females 15.7 vs. males 16.0 (ns); BF: females 13.4 vs. males 15.1 (*p* < 0.05).

## 4. Discussion

The present study offers an integrated evaluation of productive performance, carcass characteristics, meat quality, lipid composition, and muscle fibre traits in three rabbit genotypes differing in selection background and productive orientation. By adopting a slaughter-at-equal-weight approach, genotype-related biological differences were highlighted. Although the authors statistically controlled for weaning age (covariate), the pre-weaning environment (maternal effects, early nutrition) likely differed between the conventional and organic farms. In applied animal experiments dealing with local breeds, endangered genetic resources, or alternative production systems, “farm-of-origin” is a recognised structural constraint [24]. The authors acknowledge this is a possible confounding factor.

As expected, commercial hybrids exhibited superior growth performance, reaching the target slaughter weight at a younger age and with a shorter fattening period than Vienna Blue and Burgundy Fawn crosses. This outcome is consistent with previous studies reporting faster growth rates and improved feed efficiency in hybrid rabbits derived from intensive selection programmes [25,26,27]. The higher average daily gain and lower feed conversion ratio observed in commercial hybrids confirm the effectiveness of genetic selection aimed at maximising production efficiency under controlled conditions. In contrast, the slower growth rates and poorer feed efficiency recorded in BF rabbits reflect the limited selection pressure applied to local or traditional genotypes [28]. Nevertheless, the comparable efficiency between VB crosses and commercial hybrids suggests that some alternative genotypes may represent a compromise between productivity and adaptability, as also reported in comparative studies on selected and non-selected rabbit lines [29].

Importantly, sex had a negligible influence on growth traits, which aligns with the general consensus that sexual dimorphism in rabbits is modest during the growing phase [4]. The limited genotype × sex interactions further support the robustness of genotype effects across sexes.

Despite marked differences in growth dynamics, carcass yield was remarkably similar among genotypes, confirming that slower-growing rabbits can achieve comparable carcass efficiency when slaughtered at the same live weight [30]. This finding is particularly relevant for alternative production systems, where growth rate is not the sole determinant of economic sustainability.

The higher perirenal fat deposition observed in BF rabbits reflects a greater propensity for lipid storage, which has been previously associated with genotypes characterised by slower growth and later physiological maturity [9]. Diversely, the different origins of rabbits (pre-weaning period) and thus diets consumed are not considered a relevant factor to explain this finding: this is because solid feed intake in rabbits remains minimal until approximately 28–30 days of age and becomes nutritionally relevant only after weaning [31]. This trait may be viewed either as a drawback or an advantage, depending on production goals. While excessive fat deposition is undesirable in intensive systems, moderate fatness can improve meat juiciness and sensory attributes, especially in niche or traditional markets [32]. Moreover, a higher amount of lipids with beneficial effects on human health can be provided, which represents a relevant nutritional point and has a potential for market diversification strategies [33]. The heavier hind legs recorded in commercial hybrids confirm their superior muscular development, a trait commonly enhanced by selection for growth rate [34].

Meat pH values fell within the normal range for rabbit meat and were unaffected by genotype, indicating that pre-slaughter handling and post-mortem muscle metabolism were well controlled [35]. This stability supports previous observations that genotype has a limited effect on ultimate pH when slaughter conditions are standardised [36].

Although ultimate pH is a major driver of meat colour, it is not the sole determinant. When pH values fall within a narrow physiological range, as observed in the present study, differences in lightness are mainly explained by muscle microstructure and composition rather than post-mortem acidification. Larger fibres or looser fibre packing increase light scattering, and genotype-related differences in fibre morphology can therefore modify meat lightness independently of pH [37]. Moreover, muscles with a higher proportion of fast glycolytic or fast oxidative glycolytic fibres tend to appear lighter due to lower myoglobin concentration and differences in sarcoplasmic protein content, even at similar pH values [11]. Even when overall water-holding capacity is similar, differences in surface water distribution can influence reflectance, as small variations in extracellular water are sufficient to affect L* without altering pH [38]. In addition, genotype-related differences in myoglobin content or redox state can alter lightness independently of pH, particularly in muscles with mixed fibre types such as the *biceps femoris* [39]. Accordingly, the lighter colour observed in BF rabbits may be linked to differences in intramuscular fat content and muscle fibre characteristics [40].

The higher femur bone strength observed in VB rabbits, particularly in males, suggests enhanced skeletal robustness. This trait may reflect a slower growth rate and longer bone mineralization period, as described in less intensively selected genotypes [41], and represents a relevant welfare-connected characteristic.

Genotype had a limited influence on morphometric traits, fibre type distribution, and enzymatic activity of the *longissimus lumborum* muscle. The absence of significant differences in fibre cross-sectional area supports previous evidence that selection for growth performance does not necessarily induce changes in muscle fibre hypertrophy when rabbits are slaughtered at a comparable physiological stage [42,43].

Fibre type distribution was remarkably consistent across genotypes and sexes, with fast oxidative glycolytic fibres (αW) largely predominating. This pattern is typical of the *longissimus lumborum* muscle in rabbits and reflects its functional role in rapid locomotion [44]. Enzymatic activity indicators further confirmed metabolic homogeneity among genotypes. Comparable citrate synthase and lactate dehydrogenase activities indicate similar oxidative and glycolytic capacities, suggesting that differences in growth performance and carcass fat deposition are not driven by intrinsic muscle metabolic specialisation [45,46]. This metabolic uniformity is consistent with the limited genotype effects observed for physical meat traits and supports the interpretation that variations in water, lipid, and cholesterol contents of hind leg meat are mainly related to fat deposition rather than muscle fibre characteristics [47].

Accordingly, differences in water and lipid content reflect the well-established inverse relationship between these components in muscle tissue [11]. As intramuscular fat increases, water content decreases due to partial replacement of aqueous compartments by lipid droplets, a phenomenon widely documented in rabbit meat and other livestock species [29,48]. The higher lipid content observed in BF rabbits and in females agrees with reports indicating that less-selected genotypes and sex hormones influence lipid metabolism and fat deposition [28,49]. Importantly, these differences remained within physiological ranges typical of rabbit meat and are unlikely to negatively affect technological quality [28], while potentially enhancing sensory traits such as juiciness [32].

The higher cholesterol content found in commercial hybrids suggests a trade-off between production efficiency and nutritional quality, as previously reported for different rabbit genotypes [46]. The fatty acid (FA) profile of rabbit hind leg meat exhibited moderate, but biologically meaningful, variations associated with genotype and sex, which should be interpreted in close relation to the differences in lipid and cholesterol contents (Table 5). As widely documented, the level of intramuscular fat influences not only total lipid content but also the relative distribution of individual fatty acids, particularly saturated and monounsaturated fractions [29,47].

In the present study, Burgundy Fawn (BF) rabbits showed higher lipid content in hind leg meat, which was associated with a numerical increase in total saturated fatty acids (SFA) and a concomitant reduction in polyunsaturated fatty acids (PUFA). This pattern is consistent with the well-known dilution effect, whereby increasing lipid deposition leads to a relative enrichment of SFA and MUFA at the expense of PUFA due to the preferential accumulation of neutral lipids in adipose tissue [47,49]. Similar relationships between intramuscular fat content and FA composition have been reported in rabbit meat and other monogastric species [42,47].

Interestingly, the higher cholesterol content observed in commercial hybrids was not associated with increased lipid levels or higher SFA proportions, indicating that cholesterol concentration in rabbit meat is not solely dependent on intramuscular fat content. Rather, it likely reflects genotype-specific differences in lipid metabolism, membrane lipid composition, and cholesterol turnover at the muscle level [28,47]. This observation confirms that cholesterol and fatty acid profiles may vary independently and should be evaluated jointly when assessing the nutritional quality of meat.

Genotype-related differences in PUFA composition were particularly evident for linoleic acid (C18:2 *n*-6) and the *n*-6/*n*-3 ratio. Vienna Blue rabbits exhibited higher *n*-6/*n*-3 values compared with commercial hybrids, despite similar lipid contents, suggesting differences in PUFA incorporation or metabolic utilisation rather than simple fat dilution. Although the observed *n*-6/*n*-3 ratios remained within nutritionally acceptable ranges, lower values are generally regarded as more favourable for human health due to their association with reduced inflammatory and cardiovascular risk [48].

Sex effects on FA composition closely mirrored those observed for lipid content in Table 5. Females who exhibited higher intramuscular fat levels also showed increased MUFA proportions and reduced PUFA content, resulting in a higher MUFA/PUFA ratio. In contrast, males displayed higher proportions of long-chain *n*-3 PUFA, including EPA and DHA, contributing to a more favourable PUFA profile despite their lower lipid content. These patterns are consistent with previous findings highlighting the role of sex hormones in regulating lipid deposition, desaturation, and elongation pathways in rabbits [49].

Finally, these results support the concept that alternative rabbit genotypes, despite lower growth efficiency, possess valuable traits related to carcass quality and nutritional characteristics. Taken together, the integrated evaluation of lipid content, cholesterol concentration, and fatty acid composition highlights the overall nutritional suitability of rabbit meat across all genotypes. Despite some genotype and sex related differences, rabbit meat consistently exhibited high PUFA levels, moderate SFA proportions, and *n*-6/*n*-3 ratios typical for commercial diets and potentially improvable by omega-3 enrichment strategies [50], confirming its value as a lean meat with a favourable lipid profile. Importantly, alternative genotypes such as Vienna Blue and Burgundy Fawn crosses, while characterised by higher fat deposition in some cases, still produced meat with nutritional attributes compatible with health-oriented dietary recommendations. These findings reinforce the potential of rabbit biodiversity not only for sustainable production but also for delivering nutritionally valuable meat to increasingly health-conscious consumers [3,7,51].

## 5. Conclusions

The present study demonstrates that rabbit genotype markedly influences growth dynamics, carcass fat deposition, and selected nutritional traits of meat, while having a limited impact on muscle fibre characteristics and metabolic profile when animals are slaughtered at a common market weight. Commercial hybrids confirmed their superior growth rate and feed efficiency, reflecting targeted genetic selection for intensive production systems. However, this productive advantage was not consistently associated with superior carcass yield or meat quality and was accompanied by higher cholesterol content, the latter being relevant from a nutritional and, potentially, consumer preference perspective. In contrast, Vienna Blue and Burgundy Fawn crosses, despite slower growth and longer fattening periods, achieved comparable carcass yields and exhibited specific qualitative traits, including greater skeletal robustness, differentiated fat deposition patterns, and, in some cases, more favourable nutritional attributes (e.g., lower cholesterol content).

Overall, these findings highlight the strategic value of alternative rabbit genotypes to support the valorisation of rabbit biodiversity as a complementary approach to commercial hybrid production, particularly in low-input, rural, or welfare-oriented farming contexts where different attributes are prioritised over maximal growth efficiency. The integration of such genotypes may contribute to preserving genetic resources while meeting evolving consumer expectations for quality, sustainability, and nutritional value. Further research under extensive rearing conditions and including sensory evaluation will be essential to fully exploit the potential of these genetic resources.

## Figures and Tables

**Table 1:** Effects of genotype, sex and their interaction on the live performance of growing rabbits.

Items	Sire Genetic Origin (SGO)			Sex (S)		Significance				RMSE ^1^
	C	VB	BF	Female	Male	SGO	S	C vs. VB + BF	SGO × S	
N. rabbits	12	14	15	17	24					
Weaning weight, g	1079.3 ^B^	816.4 ^A^	790.0 ^A^	906.1	884.2	***	ns	***	ns	75.4
Slaughter age ^2^	88.0 ^A^	109.3 ^B^	122.1 ^C^	106.2	106.8	***	ns	***	ns	9.02
Slaughter weight, g	2863	2792	2737	2810	2785	ns	ns	*	ns	112.7
Days of fattening	48.9 ^A^	55.3 ^A^	69.6 ^B^	57.8	58.0	***	ns	***	ns	9.16
Total growth, g	1784 ^A^	1976 ^B^	1947 ^B^	1904	1901	***	ns	***	ns	97.7
Average daily gain, g/day	36.3 ^B^	36.2 ^B^	28.9 ^A^	33.9	33.7	**	ns	ns	ns	4.72
Total feed intake, g	6927 ^A^	8458 ^Ba^	9880 ^Bb^	8425	8418	***	ns	***	ns	1224
Average daily feed intake, g/day ^3^	140.2 ^A^	152.3 ^B^	152.9 ^B^	148.0	148.8	**	ns	**	*	8.90
Feed conversion ratio	3.90 ^A^	4.28 ^A^	5.02 ^B^	4.41	4.39	**	ns	**	ns	0.58

C: commercial hybrids; VB: Vienna Blue crosses; BF: Burgundy Fawn crosses; *: *p* < 0.05; **: *p* < 0.01; ***: *p* < 0.001; ^A^,^B^,^C^ Different superscript letters in a row differ for *p* < 0.01; ^a^,^b^ Different superscript letters in a row differ for *p* < 0.05; ns: not significant; ^1^ Root Mean Squared Error; ^2^ Slaughter age data are reported in days; ^3^ SGO × S interaction for average daily feed intake: C = females 139.2 vs. males 141.2 (ns); VB = females 157.8 vs. males 146.8 (*p* < 0.05); BF = females 147.2 vs. males 158.6 (*p* < 0.05).

**Table 2:** Effects of genotype, sex and their interaction on the carcass traits of rabbits.

Items	Sire Genetic Origin (SGO)			Sex (S)		Significance				RMSE ^1^
	C	VB	BF	Female	Male	SGO	S	C vs. VB + BF	SGO × S	
N. carcasses	12	14	15	17	24					
Reference carcass (RC), g	1358	1347	1298	1326	1343	ns	ns	ns	ns	72.1
Reference carcass, % CC	82.2	81.6	81.4	81.8	81.7	ns	ns	ns	ns	1.47
Perirenal fat, g	16.7 ^a^	22.6 ^a^	32.5 ^b^	23.7	24.1	*	ns	ns	ns	8.24
Scapular fat, g	8.80	8.40	8.10	8.10	8.80	ns	ns	ns	ns	2.72
Other dissectible fat, g	7.90	4.10	6.60	7.00	5.40	ns	ns	ns	ns	4.97
Total dissectible fat, g	33.4	35.0	47.2	38.8	38.3	ns	ns	ns	ns	12.9
Perirenal fat, % RC	1.22 ^A^	1.68 ^A^	2.47 ^B^	1.78	1.80	*	ns	*	ns	0.58
Scapular fat, % RC	0.66	0.62	0.63	0.61	0.66	ns	ns	ns	ns	0.20
Other dissectible fat, % RC	0.58	0.30	0.50	0.52	0.40	ns	ns	ns	ns	0.36
Total dissectible fat, % RC	2.46	2.60	3.59	2.91	2.86	ns	ns	ns	ns	0.91
Hind leg, g	232 ^AB^	230 ^B^	213 ^A^	222	228	*	ns	ns	ns	13.7
Hind leg bones, g	37.2	38.3	35.9	36.6	37.7	ns	ns	ns	ns	3.59
Hind leg meat to bone ratio (raw)	5.27	5.07	4.98	5.10	5.10	ns	ns	ns	ns	0.58
Hind legs, % RC	34.2	34.2	33.1	33.6	34.0	ns	ns	ns	ns	1.02
Loin (1st–7th lumbar vertebra), g	331	357	349	344	347	ns	ns	ns	ns	26.6
Loin, % RC	24.3 ^a^	26.5 ^b^	26.9 ^b^	25.9	25.8	*	ns	*	ns	1.53

C: commercial hybrids; VB: Vienna Blue crosses; BF: Burgundy Fawn crosses; *: *p* < 0.05; ns: not significant; ^A^,^B^ Different superscript letters in a row differ for *p* < 0.01; ^a^,^b^ Different superscript letters in a row differ for *p* < 0.05; ^1^ Root Mean Squared Error.

**Table 3:** Effects of genotype, sex and their interaction on the physical traits of rabbit *biceps femoris* and *longissimus lumborum* muscles and femur bone strength.

Items	Sire Genetic Origin (SGO)			Sex (S)		Significance				RMSE ^1^
	C	VB	BF	Female	Male	SGO	S	C vs. VB + FB	SGO × S	
N. samples	12	14	15	17	24					
*biceps femoris* (24 h *post mortem*):										
pHu	5.83	5.80	5.75	5.77	5.81	ns	ns	ns	ns	0.08
L*	50.7 ^a^	52.9 ^b^	54.1 ^bc^	52.5	52.7	*	ns	*	** ^2^	1.69
a*	4.41	4.02	3.88	4.51	3.71	ns	ns	ns	ns	1.76
b*	2.25	2.76	3.64	3.24	2.53	ns	ns	ns	ns	1.35
*longissimus lumborum* (24 h *post mortem*):										
pHu	5.66	5.64	5.65	5.63	5.67	ns	ns	ns	ns	0.07
L*	58.8	58.7	58.9	59.1	58.5	ns	ns	ns	ns	1.88
a*	2.05	2.65	1.93	2.13	2.29	ns	ns	ns	ns	1.25
b*	−2.09	−0.59	−0.08	−0.73	−1.12	ns	ns	ns	ns	1.44
Femur bone strength, kg/cm^2^:	26.7 ^ab^	30.0 ^b^	25.1 ^a^	25.0	29.4	*	*	ns	ns	4.82

C: commercial hybrids; VB: Vienna Blue crosses; BF: Burgundy Fawn crosses; *: *p* < 0.05; ^a^,^b^,^c^ Different superscript letters in a row differ for *p* < 0.05; ^1^ Root Mean Squared Error; ^2^ SGO × S: C = females 49.7 vs. males 51.8 (ns); VB = females 52.4 males 53.4 (ns); BF = females 55.3 vs. males 53.0 (*p* < 0.05).

**Table 4:** Effects of genotype, sex and their interaction on the morphometric traits, fibre type distribution and enzymatic activity of the *longissimus lumborum* muscle.

Items	Sire Genetic Origin (SGO)			Sex (S)		Significance				RMSE ^1^
	C	VB	BF	Female	Male	SGO	S	C vs. VB + BF	SGO × S	
N. samples	12	14	15	17	24					
Fibre cross-sectional area (µm^2^):										
αW	1746	1947	1555	1704	1795	ns	ns	ns	ns	538
αR	915	1215	1130	952	1221	ns	*	ns	ns	349
βR	758	1137	892	884	974	ns	ns	ns	ns	454
Fibre type distribution (%):										
αW	83.2	78.9	84.3	82.3	82.0	ns	ns	ns	ns	5.8
αR	12.9	15.4	13.2	14.1	13.5	ns	ns	ns	ns	4.2
βR	3.90	5.60	2.50	3.60	4.50	ns	ns	ns	ns	4.3
Enzymatic activity (IU) ^2^:										
CS	5.72	5.89	6.10	6.02	5.79	ns	ns	ns	ns	1.35
LDH	909	912	837	893	879	ns	ns	ns	ns	131
LDH/CS	164	163	144	155	158	ns	ns	ns	ns	39

C: commercial hybrids; VB: Vienna Blue crosses; BF: Burgundy Fawn crosses; *: *p* < 0.05; ns: not significant; ^1^ Root Mean Squared Error; ^2^ IU: moles of substrate degraded/min/g fresh meat.

**Table 5:** Effects of genotype, sex and their interaction on the proximate composition (g/100 g meat), Heme iron and cholesterol contents (mg/100 g meat) of rabbit hind leg meat.

Items	Sire Genetic Origin (SGO)			Sex (S)		Significance				RMSE ^1^
	C	VB	BF	Female	Male	SGO	S	C vs. VB + BF	SGO × S	
N. samples	12	14	15	17	24					
Water	73.0 ^ab^	73.7 ^b^	72.9 ^a^	72.9	73.5	*	*	ns	ns	0.8
Protein	22.2	21.9	22.0	21.9	22.2	ns	ns	ns	ns	0.5
Lipids	3.56 ^ab^	3.16 ^a^	3.91 ^b^	4.03	3.06	ns	***	ns	* ^2^	0.77
Ash	1.26	1.25	1.22	1.24	1.25	ns	ns	ns	ns	0.04
Heme iron	0.340	0.335	0.334	0.342	0.330	ns	ns	ns	ns	0.05
Cholesterol	65.5 ^b^	59.4 ^a^	57.8 ^a^	62.4	59.4	*	*	**	ns	3.87

C: commercial hybrids; VB: Vienna Blue crosses; BF: Burgundy Fawn crosses; *: *p* < 0.05; **: *p* < 0.01; ***: *p* < 0.001; ^a^,^b^ Different superscript letters in a row differ for *p* < 0.05; ^1^ Root Mean Squared Error; ^2^ SGO × S: C = females 4.14 vs. males 2.98 (*p* < 0.05). VB = females 3.16% vs. males 3.15% (ns); BF = females 4.78 vs. males 3.04 (*p* < 0.001).

**Table 6:** Effects of genotype, sex and their interaction on the fatty acids (FA) profile (% total fatty acids methyl esters) of rabbit hind leg meat.

Items	Sire Genetic Origin (SGO)			Sex (S)		Significance				RMSE ^1^
	C	VB	BF	Female	Male	SGO	S	C vs. VB + BF	SGO × S	
N. samples	12	14	15	17	24					
C6:0	0.059	0.133	0.157	0.049	0.183	ns	*	ns	ns	0.191
C8:0	0.0003 ^a^	0.032 ^b^	0.009 ^a^	0.023	0.005	**	*	ns	ns	0.023
C10:0	0.049 ^ab^	0.042 ^b^	0.009 ^a^	0.032	0.035	*	ns	ns	ns	0.028
C12:0	0.069	0.074	0.066	0.071	0.068	ns	ns	ns	ns	0.039
C14:0	2.16	2.18	2.28	2.40	2.02	ns	***	ns	ns	0.291
C15:0	0.49	0.479	0.446	0.481	0.463	ns	ns	ns	ns	0.085
C16:0	28	28.8	30.1	29.2	28.7	ns	ns	ns	ns	1.882
C17:0	0.746	0.711	0.652	0.706	0.700	ns	ns	ns	ns	0.057
C18:0	7.58	8.12	8.55	7.86	8.31	ns	ns	ns	ns	0.720
C20:0	0.112	0.125	0.120	0.12	0.118	ns	ns	ns	ns	0.022
C21:0	0.118	0.174	0.227	0.143	0.203	ns	ns	ns	ns	0.319
Total saturated FA (SFA)	39.4	40.9	42.5	41.1	40.7	ns	ns	ns	ns	2.23
C14:1	0.109	0.120	0.127	0.152	0.086	ns	**	ns	ns	0.059
C16:1	2.79	2.67	3.05	3.17	2.51	ns	**	ns	ns	0.626
C17:1	0.244	0.276	0.242	0.241	0.267	ns	ns	ns	ns	0.065
C18:1 *n*-9	21.9	21.5	21.3	22.2	20.9	ns	*	ns	ns	1.664
C18:1 *n*-7	1.40	1.40	1.36	1.31	1.46	ns	***	ns	ns	0.110
C20:1 *n*-9	0.228	0.211	0.192	0.213	0.208	ns	ns	ns	ns	0.043
C24:1 *n*-9	<0.0001	0.010	0.018	0.006	0.010	ns	ns	ns	ns	0.022
Total monounsaturated FA (MUFA)	26.7	26.3	26.0	27.3	25.4	ns	*	ns	ns	2.28
C18:2 *n*-6	28.8 ^b^	26.9 ^b^	25.4 ^a^	26.7	27.4	*	ns	*	ns	1.612
C18:3 *n*-6	0.066	0.047	0.028	0.052	0.043	ns	ns	ns	ns	0.023
C18:3 *n*-3	1.95	1.62	1.50	1.81	1.57	ns	*	*	ns	0.283
CLA + C18:4 *n*-3	0.032	0.069	0.069	0.054	0.060	ns	ns	ns	ns	0.032
C20:2 *n*-6	0.303	0.364	0.339	0.302	0.369	ns	*	ns	ns	0.085
C20:3 *n*-6	0.204	0.303	0.303	0.216	0.325	ns	**	ns	ns	0.102
C20:4 *n*-6	2.06	3.17	3.34	2.13	3.58	ns	**	ns	ns	1.233
C20:3 *n*-3	0.106	0.109	0.116	0.076	0.144	ns	ns	ns	ns	0.174
C20:5 *n*-3 (EPA)	0.010	0.045	0.054	0.024	0.048	ns	*	ns	ns	0.031
C22:5 *n*-3	0.263	0.243	0.339	0.164	0.400	ns	***	ns	ns	0.181
C22:6 *n*-3 (DHA)	0.036 ^A^	0.101 ^B^	0.067 ^AB^	0.050	0.086	**	**	ns	ns	0.038
Total polyunsaturated FA (PUFA)	33.9	32.8	31.4	31.6	33.8	ns	*	ns	ns	2.49
SFA/UFA	0.654	0.693	0.738	0.700	0.690	ns	ns	ns	ns	0.065
MUFA/PUFA	0.796	0.811	0.842	0.869	0.763	ns	**	ns	ns	0.105
*n*-6	31.4	30.7	29.3	29.4	31.5	ns	**	ns	ns	2.320
*n*-3	2.37	2.04	2.07	2.13	2.20	ns	ns	*	ns	0.236
*n*-6/*n*-3	13.3 ^a^	15.1 ^b^	14.2 ^ab^	14.0	14.4	*	ns	ns	* ^2^	1.46

C: commercial hybrids; VB: Vienna Blue crosses; BF: Burgundy Fawn crosses; *: *p* < 0.05; **: *p* < 0.01; ***: *p* < 0.001; ^A^,^B^ Different superscript letters in a row differ for *p* < 0.01; ^a^,^b^ Different superscript letters in a row differ for *p* < 0.05; ^1^ Root Mean Squared Error; ^2^ SGO × S: C = females 13.0 vs. males 13.6 (ns); VB = females 15.7 vs. males 16.0 (ns); BF = females 13.4 vs. males 15.1 (*p* < 0.05).

## Data Availability

Dataset available on request from the authors: the raw data supporting the conclusions of this article will be made available by the authors on request.
